# Surgery-induced wound response promotes stem-like and tumor-initiating features of breast cancer cells, *via* STAT3 signaling

**DOI:** 10.18632/oncotarget.2195

**Published:** 2014-07-09

**Authors:** Ilenia Segatto, Stefania Berton, Maura Sonego, Samuele Massarut, Tiziana Perin, Erica Piccoli, Alfonso Colombatti, Andrea Vecchione, Gustavo Baldassarre, Barbara Belletti

**Affiliations:** ^1^ Division of Experimental Oncology 2, CRO, National Cancer Institute, Aviano, Italy; ^2^ Breast Surgery Unit, CRO, National Cancer Institute, Aviano, Italy; ^3^ Pathology Unit, CRO, National Cancer Institute, Aviano, Italy; ^4^ Department of Scienze Biologiche e Mediche, MATI Center of Excellence, University of Udine, Udine, Italy; ^5^ Division of Pathology, II University of Rome “La Sapienza”, Santo Andrea Hospital, Rome, Italy

**Keywords:** STAT3, breast cancer, cancer stem cells, tumor initiating cells, CSC-like properties

## Abstract

Inflammation is clinically linked to cancer but the mechanisms are not fully understood. Surgery itself elicits a range of inflammatory responses, suggesting that it could represent a perturbing factor in the process of local recurrence and/or metastasis formation.

Post-surgery wound fluids (WF), drained from breast cancer patients, are rich in cytokines and growth factors, stimulate the *in vitro* growth of breast cancer cells and are potent activators of the STAT transcription factors. We wondered whether STAT signaling was functionally involved in the response of breast cancer cells to post-surgical inflammation. We discovered that WF induced the enrichment of breast cancer cells with stem-like phenotypes, via activation of STAT3. *In vitro*, WF highly stimulated mammosphere formation and self-renewal of breast cancer cells. *In vivo*, STAT3 signaling was critical for breast cancer cell tumorigenicity and for the formation of local relapse after surgery.

Overall, we demonstrate here that surgery-induced inflammation promotes stem-like phenotypes and tumor-initiating abilities of breast cancer cells. Interfering with STAT3 signaling with a peri-surgical treatment is sufficient to strongly suppress this process. The understanding of the crosstalk between breast tumor-initiating cells and their microenvironment may open the way to successful targeting of these cells in their initial stages of growth and be eventually curative.

## INTRODUCTION

Breast cancer (BC) represents the most common malignancy in women worldwide. To date, localized disease is largely curable, while disseminated and systemic disease is what mostly influences patients' outcome [1]. However, local relapse represents the single most important risk factor and an independent prognostic factor for survival of BC patients and if rates of local recurrence could be minimized in the first 5-years post-surgery, this will eventually impact on overall survival of BC patients [1, 2]. Thus restraining this pathological event represents a compelling objective in breast cancer research.

Many evidences in literature indicate that the stem-like phenotype of cancer cells may account for their capability to lead to disease relapse, even when the primary tumor is eradicated [3]. These tumor-initiating cells (TIC) share properties of self-renewal and differentiation with normal stem cell counterparts, although in TIC these processes are deregulated [3].

Despite the fact that most of BC is multicentric, 90% of local recurrences occur at the same quadrant of the primary cancer [1]. It is well known that inflammation activates positive feedback loops that allow the maintaining of a transformed state even in the absence of the inducing signals. Surgery itself elicits a range of inflammatory responses which are known to modify the growth kinetics of breast cancer micro metastasis [4], suggesting that it could represent a perturbing factor in the process of local recurrence and/or metastasis development in humans, as already demonstrated in animal models [5, 6]. The molecular events associated with the wound healing process could provide a sort of ‘start signal’ for the awakening of existing tumor foci in the ipsilateral quadrant or of dormant disseminated micro metastases. In accord with these observations, risk of local recurrence remains high mainly in the area surrounding the original tumor and particularly along the scar, suggesting that the wound healing process that follows surgery may be implicated [1]. This clinical observation has stimulated the research of novel peri-surgical treatments, aimed to both killing residual tumor cells and affecting the tumor microenvironment.

From a molecular point of view, we recently tested the hypothesis that modification of the local microenvironment by surgery may alter the growth kinetics of BC cells [7]. One signaling pathway significantly induced in BC cells was the Signal Transducer and Activator of Transcription 3 (STAT3) pathway [7]. Virtually every cytokine and growth factor can stimulate STAT3 activation, by receptor induced tyrosine phosphorylation, followed by dimerization and nuclear translocation. In the nucleus, active STAT3 recognizes specific DNA sequences and results in transcription of genes that control critical cellular functions including cell proliferation, survival and self-renewal but also cell differentiation and apoptosis [8]. It has been proposed that cancer cells displaying stem-like features play a critical role in the onset of local relapse, propagation of metastasis, as well as in drug resistance. Accumulating evidences support a role for STAT3 pathway in cancer stem cell functions, particularly in breast tumor-initiating cells [9]. Despite the fact that a sizable body of evidences highlight that STAT3 is inappropriately activated in a vast percentage of breast tumors, its biological significance is controversial and its concrete role in breast cancer initiation and/or progression is not fully established [10-12]. Clinical, epidemiological and molecular studies support a strong association between inflammation and cancer, as well as between STAT3 and inflammation [13-15].

In this work, we aimed to investigate the tumor microenvironment in the post-surgical setting. Our findings highlight that the inflammatory response induced by surgery may engage STAT3 pathway activation in residual BC cells and this, in turn, leads to the acquisition of stem-like features. These results could provide new therapeutic options to restrain breast cancer relapse, such as the exploitation of STAT3 inhibitors as peri-surgical treatment of BC patients.

## RESULTS

### Wound Fluids collected from breast cancer patients after surgery strongly induce mammosphere formation in BC cells

**Figure 1:**
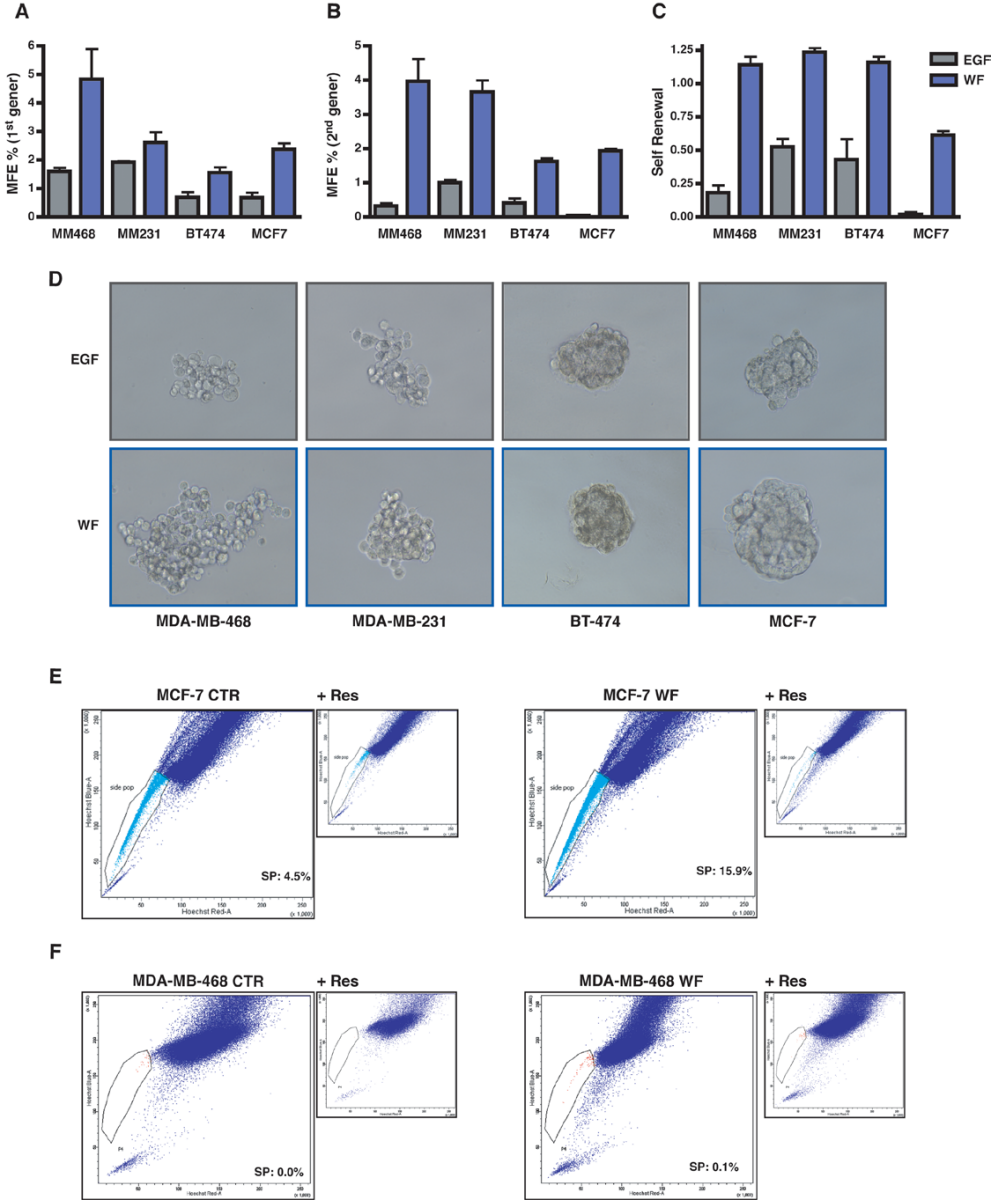
Wound Fluids stimulate growth and self-renewal of tumor initiating cells (A) Graph reports primary generation mammosphere forming efficiency (MFE) in MDA-MB-468, MDA-MB-231, BT-474 and MCF-7 cells. Cells were plated as single suspension on poly-HEMA coated dishes in mammosphere standard medium containing EGF or supplemented with 5% wound fluids (WF) and no EGF. MFE was calculated as the ratio between the number of mammospheres and the cells seeded *per* well. (B) Same as in (A), but on secondary generation mammosphere, *i.e.* mammospheres formed by cells dissociated and replated as single cells in the indicated medium from primary generation mammospheres. (C) Self-renewal in MDA-MB-468, MDA-MB-231, BT-474 and MCF-7 cells. Self-renewal was calculated as the ratio between numbers of secondary and primary mammospheres. (D) Representative pictures of the mammospheres formed by MDA-MB-468, MDA-MB-231, BT-474 and MCF-7 cells in the primary generation. (E) FACS analysis for evaluation of side population (SP) in MCF-7 cells grown in complete medium (MCF-7 CTR) or in serum free medium supplemented with 5% WF for 48 hours (MCF-7 WF). SP is identified through exclusion of Hoechst dye that is inhibited in the presence of Reserpine. Percentage of SP is reported inside the plot. (F) Same as in (E) but using MDA-MB-468 cells.

Our previous studies highlighted that wound fluids (WF), collected for 24 hours from breast cancer(BC) patients after surgery, played a prominent role in BC cells proliferation, motility and survival [7, 16, 17]. We hypothesized that the post-surgical setting and the wound healing process may also stimulate the growth and/or the survival of BC cells with tumor-initiating features. Tumor-initiating cells (TIC) are considered responsible for tumor formation and progression and, interestingly, they are endowed with stem/progenitor cell properties; in particular, tumor-initiating cells share with stem cells the key feature of self-renewal. To test this hypothesis, we used WF to support the growth of BC cells grown as suspension sphere cultures (*i.e.* mammospheres). Mammosphere cultures have been used to characterize, enrich for and propagate BC cells with stem-like or tumor-initiating phenotypes. Using EGF, as standard stimulating agent for mammosphere formation, or WF, we tested the mammosphere forming efficiency (MFE) of BC cell lines corresponding to different pathological subtypes, such as basal (MDA-MB-468 and MDA-MB-231), luminal (MCF-7) and HER-2 positive (BT-474). All tested cell lines responded to WF stimulation with a MFE higher than the one obtained with EGF (Figure 1A). Stem cells are mainly defined by their ability to self-renew, which is assessable *in vitro* by measuring the ability of mammosphere-derived cells to form new spheres. Our experiments highlighted a strong stimulating effect of WF in the self-renewal potential of BC cell lines (Figure 1B and C). Moreover, mammospheres derived from WF-stimulated BC cells were bigger in size and with higher degree of cellularity (Figure 1D).

A characteristic shared by many adult stem cells is the ability of these cells to exclude dyes, such as rhodamine and Hoechst [18]. This property, blocked by the nonspecific inhibitor of membrane transport Reserpin, identifies a small subset of cells termed the side population (SP) enriched in tumor initiating, stem-like cancer cells. We exploited this approach to corroborate the hypothesis that WF stimulated the enrichment in TIC. FACS analysis revealed that prolonged stimulation with WF strongly increased the percentage of side population in MCF-7 cells, passing from 4.5% to 15.9% (Figure 1E). The same was true also for MDA-MB-468 cell line, although these cells display much lower percent of side population (Figure 1F). Thus, our results clearly demonstrate that WF collected from BC patients after surgery contain factors that are highly stimulatory of the self renewal and stem-like phenotypes of BC cells.

### WF strongly activate STAT3 in breast cancer cell lines

**Figure 2:**
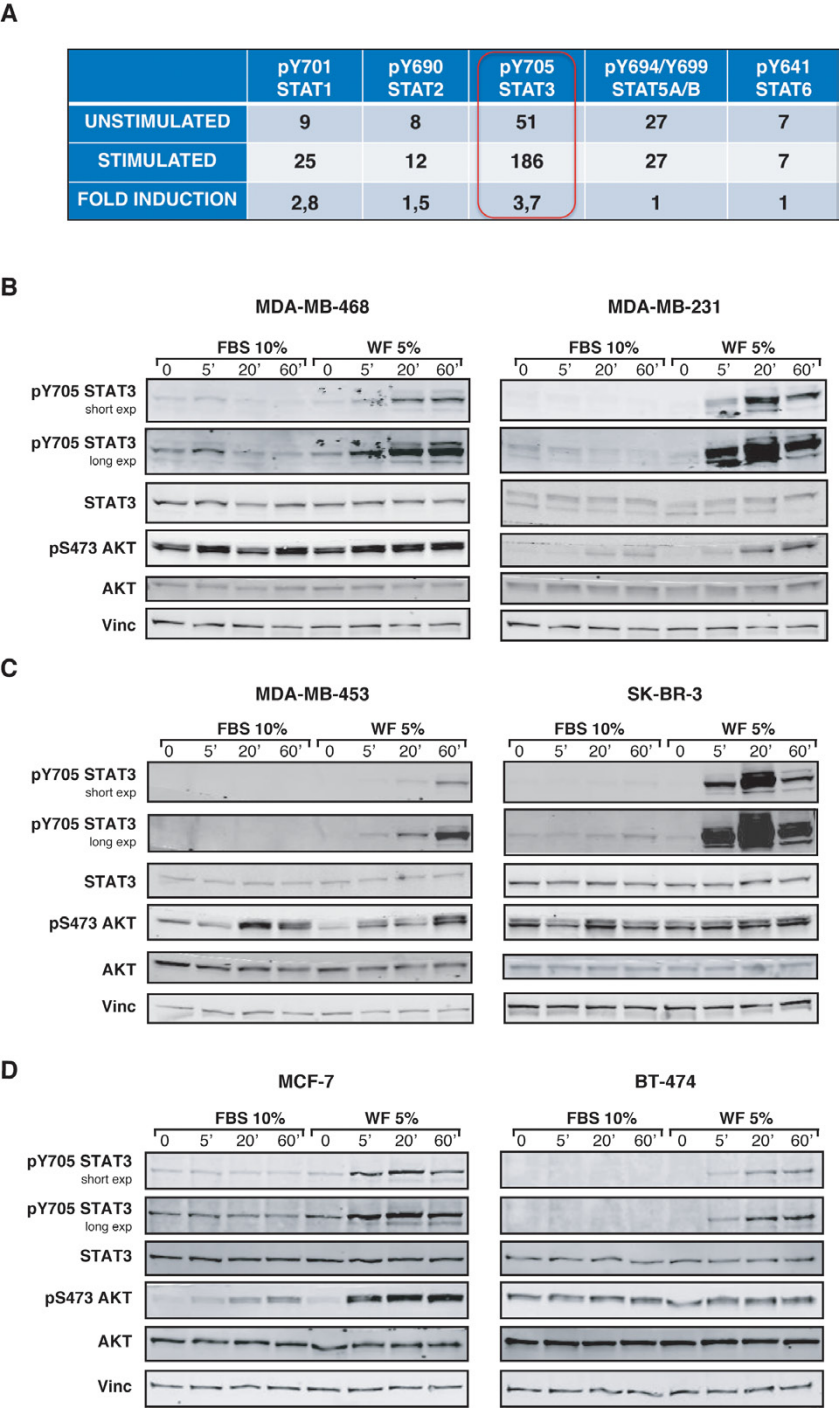
STAT3 is strongly activated in breast cancer cells, following stimulation with Wound Fluids (A) Table reports the activation of five different members of STAT family in MDA-MB-231 cells, following stimulation with 5% wound fluids for 20 minutes (stimulated) or not (unstimulated). Activation was detected using a commercial immunoassay. The value of “fold induction” represents the ratio between the unstimulated and stimulated values. (B) Western blot analysis of MDA-MB-468 and MDA-MB-231 cell lines serum starved and then stimulated for the indicated times with 10% serum (FBS) or 5% wound fluids (WF). (C) Same as in (A), but using MDA-MB-453 and SK-BR-3 cell lines, as indicates. (D) Same as in (A), but using MCF-7 and BT-474 cell lines, as indicated. Vinculin expression was used as loading control.

The role of STAT3 signaling pathway in the stem-like phenotypes of BC cells has been thoroughly described [9, 19-22]. Moreover, it is well known that, particularly in the inflammatory setting, the activation of cytokine receptors/JAK/STAT3-axis plays a primary role in the crosstalk between tumor stroma and cancer cells, eventually driving tumor progression [11, 15]. In our previous work, we demonstrated that WF stimulated BC cell proliferation and motility and also suggested that activation of STAT3 pathway might be involved in the acquisition of those phenotypes [7]. STAT3 belongs to a family of signal transducers and transcription factors, thus we first evaluated the ability of WF to activate any of the STAT family members. As indicated in the table (Figure 2A), this assay confirmed that STAT3 was strongly activated in BC cells following stimulation with WF, evaluated both as absolute level and as fold induction respect to the unstimulated cells (3.7x, Figure 2A). Among the other STAT proteins analyzed, STAT1 was also efficiently activated, but to a much lesser extent respect to STAT3. Using a large panel of BC cells, we next analyzed STAT3 activation in time course experiments with WF stimulation. In all tested cell lines, WF used at only 5% in medium induced a highly specific and sustained STAT3 activation (Figure 2B-D). Moreover, we evaluated STAT3 activation at longer time points (2-24 hours after WF stimulation), to verify whether its activation was reflected by an increased transcription of its targets. Our experiments revealed that STAT3 activation remained high also after long exposure to WF (Figure S1A and C). Although at different extent among different cell lines and at moderate levels, also the expression of STAT3 targets, such as Bcl2, cyclin D1 and survivin, was augmented, both at protein (Figure S1A and C) and RNA level (Figure S1B and D). Thus, our results confirm that WF stimulate a strong and sustained activation of STAT3 signaling activity in BC cells.

### STAT3 signaling impacts on proliferative phenotype of BC cells

**Figure 3:**
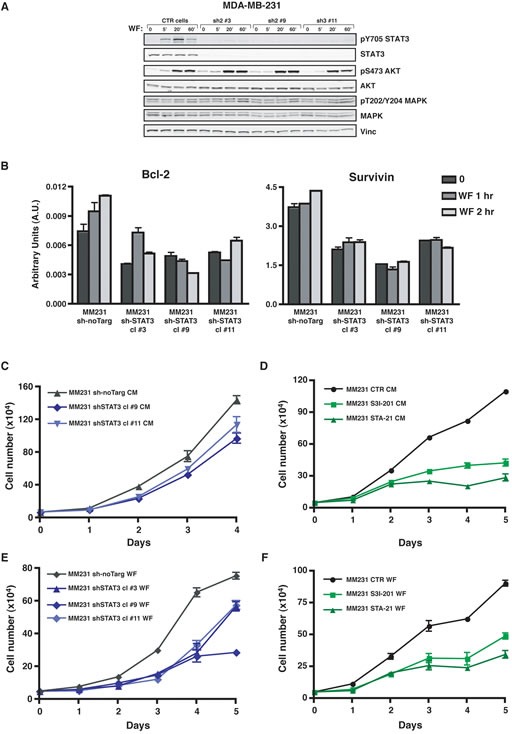
Activation of STAT3 following Wound Fluid stimulation is efficiently impaired in breast cancer cells modified for STAT3 expression (A) Western blot analysis of MDA-MB-231 cell line stably transduced with a lentiviral vector encoding for control sh-RNA (CTR) or for sh-RNAs directed against human STAT3 (sh), serum starved and then stimulated for the indicated times with 5% wound fluids (WF). Vinculin expression was used as loading control. (B) qRT-PCR analysis of Bcl-2 and Survivin expression in MDA-MB-231 control cells (sh-no Target) or in STAT3 silenced clones (sh-STAT3). Cells were serum starved and then stimulated for the indicated times with wound fluids (WF). Data represents the mean (± S.D.) of two independent experiments performed in triplicate. (C) Growth curve analysis of MDA-MB-231 control cells (sh-no Target) or STAT3 silenced clones (sh-STAT3). Cells (50×10^3^/well) have been seeded in complete medium (CM) on day 0, and then counted by Trypan Blue exclusion test, every day for 5 days. Two independent cell clones have been evaluated. Data represents the mean (± S.D.) of two independent experiments performed in triplicate. (D) Growth curve analysis of MDA-MB-231 cell line in the presence of the indicated inhibitors. Cells (50×10^3^/well) have been seeded in complete medium (CM) on day 0, in the presence of S3I-201 (50 μM) or STA-21 (30 μM) or vehicle (CTR) and then counted by Trypan blue exclusion test, every day for 5 days. Fresh medium containing the inhibitor was replaced on day 3. Data represents the mean (± S.D.) of two independent experiments performed in triplicate. (E) Same as in (C), but seeding cells in serum free medium supplemented with 3% wound fluids (WF). Three independent cell clones have been evaluated. Data represents the mean (± S.D.) of two independent experiments performed in triplicate. (F) Same as in (D), but seeding cells in serum free supplemented with 3% wound fluids (WF). Data represents the mean (± S.D.) of two independent experiments performed in triplicate.

To investigate whether activation of STAT3 signaling pathway had potential biological consequences in the response of BC cells to WF, we generated different approaches aimed to diminish STAT3 activity in BC cells. We either silenced STAT3 expression (sh-STAT3) (Figure 3A and Figure S2A) or used a panel of commercially available inhibitors (Figure S3A and C). In all conditions, the actual efficiency in decreasing STAT3 expression or activation, measured by evaluation of pY705 and/or nuclear translocation was controlled and established *in vitro* (Figure 3A, Figures S2A and S3A and C). As a control, we also verified that impairment of STAT3 did not interfere with the WF-dependent activation of other relevant proliferation and/or survival pathways such as AKT and MAPK (Figure 3A and Figure S2A). Many commercially available inhibitors were tested, but only those that were contextually able to lower the expression of STAT3 target genes were chosen for the subsequent experiments. In this context, many inhibitors were discarded for their inefficacy in inhibiting the transcription of STAT3 target genes (not shown). Suppression of STAT3 signaling, by means of specific sh-RNA, efficiently led to reduced transcription of target genes, such as Bcl2, survivin and cyclin D1 (Figure 3B and Figure S3B and D). Not surprisingly and in line with many literature data, STAT3 silencing or inhibition strongly affected the proliferative behavior of BC cell lines, when grown either in complete medium (CM, Figure 3C and D and Figure S2B and C) or in presence of WF (Figure 3E and F and Figure S2D and E). Our data thus confirm that STAT3 activity is primarily involved in proliferation of BC cells. But, more interestingly, our results also indicate that WF contain stimuli that trigger proliferation and/or survival *via* activation of STAT3 signaling pathway.

### STAT3 signaling is necessary for WF-induced self-renewal of BC cells

**Figure 4:**
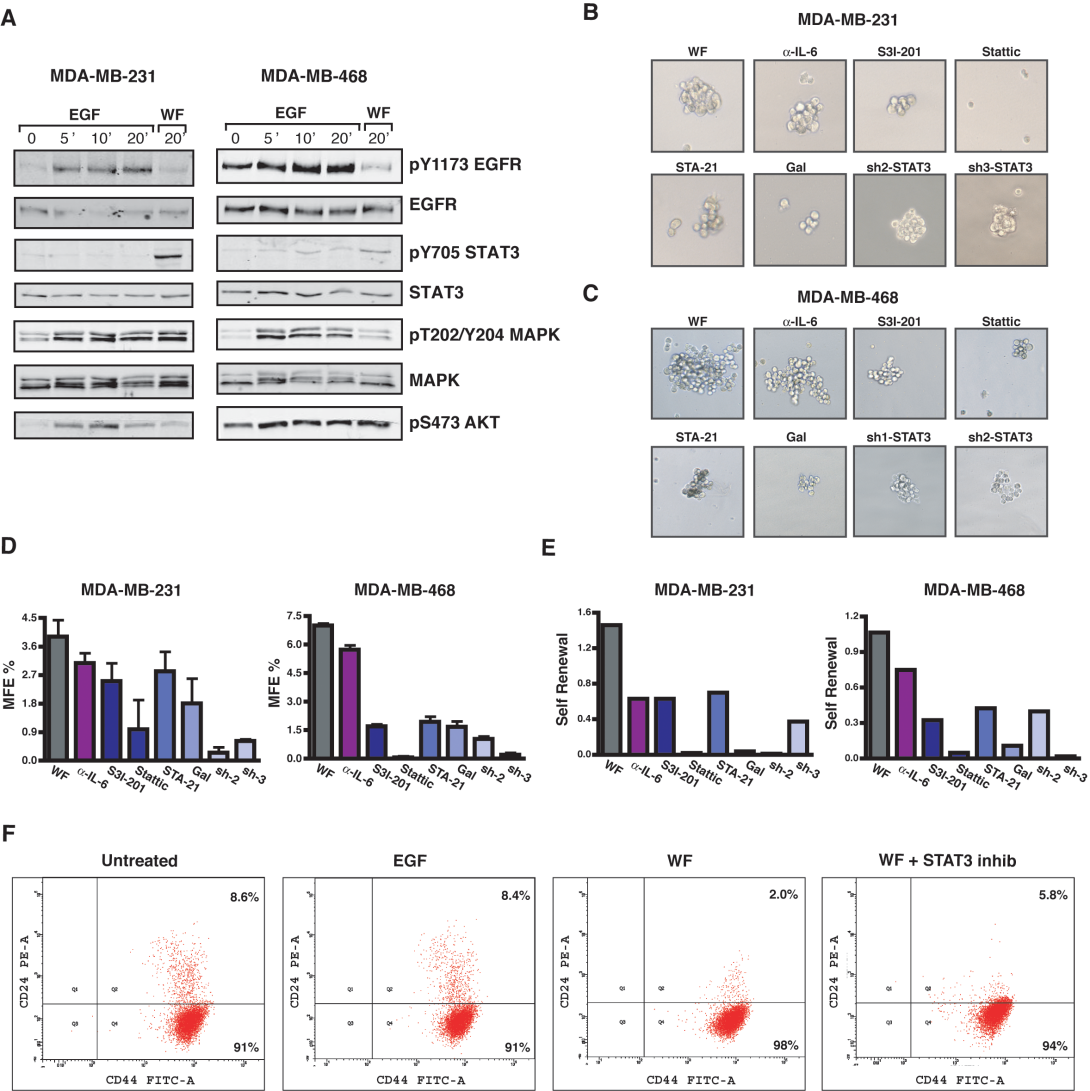
Inhibition of STAT3 impacts on growth and self-renewal of tumor-initiating cells (A) Western blot analysis of MDA-MB-231 and MDA-MB-468 cell lines, serum starved and then stimulated for the indicated times with EGF (20 ng/mL) or 5% wound fluids (WF), as indicated. (B) Images show primary mammospheres formed by MDA-MB-231 cells. Control or STAT3-silenced (sh2 and sh3) cells were plated on poly-HEMA coated dishes in mammosphere growing medium supplemented with 5% wound fluids, in the presence of IL-6 blocking antibody (0.2μg/ml) or STAT3 inhibitors (S3I-201, 50 μM; Stattic, 10 μM; STA-21, 30 μM; and Galiellalactone, 12 μM), as indicated, and grown for ten days. (C) Same as in (B) but using MDA-MB-468 cells. STAT3 inhibitors were used as follows: S3I-201, 100 μM; Stattic, 10 μM; STA-21, 30 μM; Galiellalactone, 25 μM. (D) Graphs report the percent of mammosphere forming efficiency (MFE%) in MDA-MB-231 (left) and MDA-MB-468 (right) cells of the experiment described in (B) and (C). MFE was calculated as the ratio between the numbers of mammospheres counted/number of cells seeded, *per* well. (E) Graphs report the self-renewal in MDA-MB-231 (left) and MDA-MB-468 (right) cells treated with the inhibitors only during the second generation. Self-renewal was calculated as the ratio between number of secondary mammospheres/number of primary mammospheres. (F) Flow cytometry analysis of CD44^high^CD24^low/neg^ stem cell-like subpopulation in MDA-MB-231 cells. Percent of CD44^high^CD24^low/neg^ (Q4) and of CD44^high^CD24^high^ (Q2) is reported in the plots.

Our observation that WF triggered acquisition of stem-like properties and strongly stimulated activation of STAT3 in BC cells suggested that these events could be functionally related, as proposed also by recent works [9]. Accordingly, the comparison of STAT3 activation levels elicited by WF respect to EGF stimulation clearly demonstrated that STAT3 was very specifically involved in the response of BC to WF, while much less activated by EGF (Figure 4A). To better verify the hypothesis that STAT3 critically mediated the effects of WF on BC self-renewal, we evaluated the MFE of BC cell lines, in the presence of WF stimulation, in presence or not of STAT3 inhibitors. We have previously demonstrated that IL-6 is abundantly present in WF [7] and it is known that IL-6 triggers many of the STAT3-related phenotypes [15, 19]. We thus tested whether mammospheres formation and STAT3 activation were mediated by IL-6 signaling in this setting. To this aim, we treated BC cells also with an IL-6 blocking antibody. Although at different extent, all STAT3 inhibitors considerably restrained mammosphere formation in BC cells (Figure 4B, C and D). More importantly, none of the inhibitor-treated first-generation mammospheres was able to engender second-generation mammospheres, thus indicating that the self-renewal potential of these cells was strongly suppressed by STAT3 inhibition (not shown). To better establish the role of STAT3 in the self-renewal of BC cells we used first-generation mammospheres from untreated cells and we treated cells with STAT3 inhibitors only during the second-generation (Figure 4E). Also using this mitigated condition, all inhibitors elicited a marked decrease in the ability to engender second-generation mammospheres, thus confirming that STAT3 activity was critical for the WF-induced self-renewal of BC cells. It is interesting to note that the use of the IL-6 blocking antibody elicited a decrease of MFE, but failed to induce a suppression comparable to that obtained with STAT3 inhibition (Figure 4B-E). This result indicated that, although IL-6 is present and active in the WF [7] it did not represent the principal mediator of STAT3 activation in this setting nor was it the only/primary cytokine mediating the WF potential to stimulate stem-like phenotypes in BC cells. As a further confirmation of our findings, we also evaluated the expression of surface marker profiles associated with breast cancer stem-like phenotypes, in particular the CD44^high^/CD24^low/neg^ profile [9, 19]. The presence of WF induced the reduction of the CD44^high^/CD24^high^ subpopulation of MDA-MB-231 cells, causing the increase of the CD44^high^/CD24^low/neg^ surface markers, associated with breast cancer stem cells subpopulation (Figure 4F). STAT3 inhibition partially prevented this process and cells displayed an overall increase of CD24 expression (Figure 4F, right panel).

### STAT3 activity strongly impacts on growth of primary breast tumors

**Figure 5:**
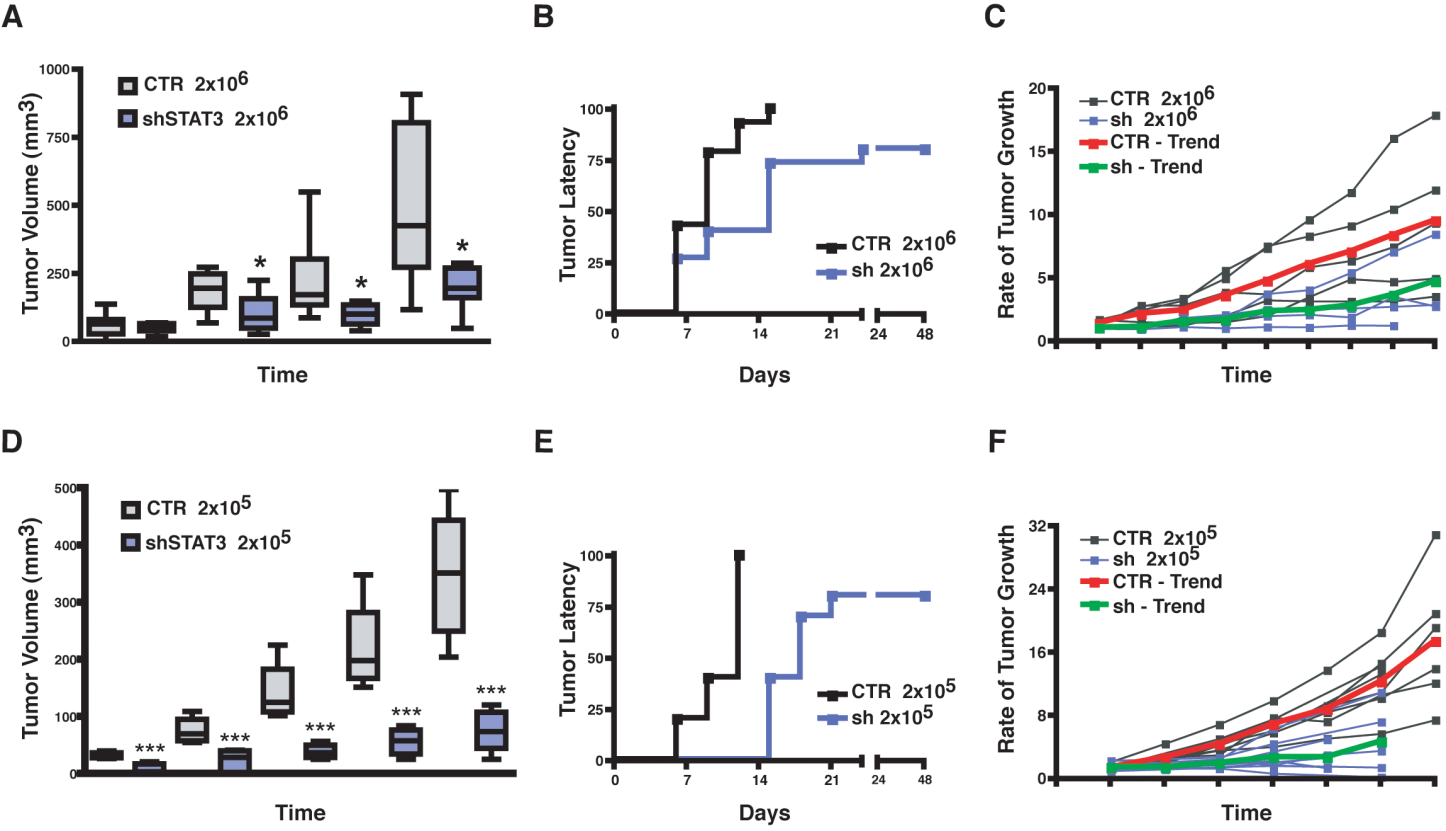
Silencing of STAT3 increases latency and decreases growth of primary breast tumors (A) Graph reports the volume (mm^3^) of primary tumors derived from injection of 2×10^6^ MDA-MB-231 control (CTR) or STAT3 silenced (sh-STAT3) cells in thoracic mammary fat pads of nude mice (2 MFP/mouse) in 50 μl Matrigel/PBS (1:1). (B) Graph reports the time dependent appearance of primary tumors derived from injection of 2×10^6^ MDA-MB-231 control (CTR) or STAT3 silenced (sh-STAT3) cells in the nude mouse thoracic mammary fat pads (2 MFP/mouse) in 50 μl Matrigel/PBS (1:1). (C) Graph reports the rate of tumor growth, independently from the time of appearance, in mice described in (A). Values are expressed as ratio of the tumor volume over the value of 20 mm^3^, considered as cut off. The red and the green lines represent the trend of growth of the MDA-MB-231 CTR and sh-STAT3, respectively. (D) Same as in (A), but injecting 2×10^5^ MDA-MB-231 (CTR) or STAT3 silenced (sh-STAT3) cells, in place of 2×10^6^ cells. (E) Same as in (B), but injecting 2×10^5^ cells. (F) Same as in (C), but injecting 2×10^5^ cells. In all graphs, statistical significance was calculated using the Student's t-test. One asterisk (*) indicates a p value ≤ 0.05, two asterisks (**) a p value ≤ 0.01 and three asterisk (***) a p value ≤ 0.005.

So far, our data indicated that WF strongly induced stem-like phenotypes of BC cells, mainly relying on STAT3 signaling. We were thus keen to evaluate the *in vivo* consequences of these findings. Constitutive activation of STAT3 is involved in the formation of a variety of different tumors, including breast cancer [23]. To characterize the role of STAT3 in our model system we first tested the ability of BC cells to grow *in vivo* when STAT3 signaling was silenced. We bilaterally injected BC cells in the fat pads of the thoracic mammary glands (MFP) of nude mice using Matrigel to support their initial survival and waited for the primary tumors to grow (2×10^5^, 7.5×10^5^ and 2×10^6^ cells/MFP). In line with reported studies [23] the suppression of STAT3 activity in BC cells strongly impacted on primary tumor growth and STAT3-silenced cells grew significantly less than control cells (Figure 5A and, Figure S4). Closer observation of tumor growth rates indicated that impairment of STAT3 signaling impacted in a dual manner in BC tumor growth. On one side it increased the tumor latency (Figure 5B and E), raising from 6 to 15 days in control *vs* STAT3-silenced cells, when injected at 2×10^5^; on the other, the measurement of tumor growth rates once that the tumors started to grow revealed that STAT3-silenced cells displayed a significantly slower proliferation rate than control cells (Figure 5C and F). These observations pointed out that, in the process of tumor initiation, STAT3 signaling plays a role in both the latency and the proliferation of BC cells.

### STAT3 signaling is critical for the growth of breast tumor initiating cells

**Figure 6:**
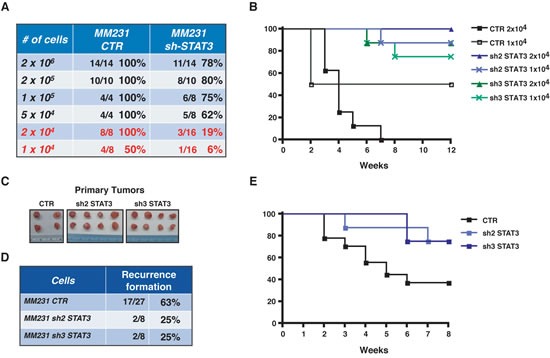
STAT3 activity impacts on breast tumor take-rate and recurrence formation (A) Table reports the percentage of tumor take-rate, following injection of the indicated numbers of MDA-MB-231 CTR or sh-STAT3 clones, in the presence of Matrigel. The media between 2 to 3 different sh-STAT3 clones is reported. (B) Graph reports the disease free survival in the indicated cohorts of mice, after injection of decreasing number of MDA-MB-231 CTR or two different sh-STAT3 clones. Data are reported as percentage of mice that developed primary tumors during 12 weeks of follow-up. (C) The picture shows an example of primary masses excised from nude mice after surgery. It is evidenced the similar dimension of the tumors in the different groups. (D) Table reports the percentage of local recurrences formed by mice injected with MDA-MB-231 CTR cells or two different clones of sh-STAT3 after 8 weeks of follow up. (E) Graph reports the disease free survival in the indicated cohorts of mice, after removal of the primary tumor. Data are reported as percentage of mice that developed recurrent disease during 8 weeks of follow-up.

Interestingly, although relatively high numbers of BC cells were injected (2×10^5^, 7.5×10^5^ and 2×10^6^ cells/MFP) a small but reproducible difference in the ability of BC to initiate the growth of a tumor mass was appreciable in all conditions (Figure 5B and E and Figure 6A, upper lines of the Table). To better discriminate among the effects exerted by STAT3 on survival and/or proliferation of BC cells, we scaled down the number of BC cells injected into nude mice MFP and evaluated the tumor take rate, hypothesizing that, under this condition, the role of STAT3 in inducing the tumor-initiating features of BC cells would be prominent. To exclude that we omitted tumor appearances due to the decreased proliferation rate of STAT3-silenced cells, we performed long tumor follow-up (10 weeks). To exclude clonal variability, we used different clones of MDA-MB-231 cells (Figure 3A) expressing different sequences to silence STAT3. Results from these *in vivo* experiments clearly showed that while control cells initiated tumors in 100% (2×10^4^) and 50% (1×10^4^) of injection sites, STAT3-silenced cells initiated a tumor only in 19% and 6% of the cases, respectively (Figure 6A and B). These data demonstrate that an intact STAT3 signaling is critically necessary for tumor initiation of BC cells.

### Silencing STAT3 in breast cancer cells decreases the rate of local recurrence

The above results confirmed in our model system an already well-established role of STAT3 in tumor initiation [21]. Our major goal was to establish whether these findings were implicated in pathological contexts related to post-surgical inflammation. We hypothesized that residual cancer cells in the post-surgery setting may respond to inflammatory stimuli, enriching for tumor-initiating cells via STAT3 signaling. To test this hypothesis, we evaluated local recurrence after surgery, as read out of tumor initiating ability, in control- and STAT3-silenced BC cells. Following surgical removal of primary tumors, grown at similar size (Figure 6C), we evaluated the rate of loco-regional relapse using a mouse model for breast cancer recurrence recently set up in our laboratory [16]. Post-surgical follow up of 8 weeks clearly showed that silencing of STAT3 impacted on local recurrence of BC, diminishing the percent of recurrence from 63% to 25% (Figure 6D and E). Altogether these findings indicate that impairment of STAT3 activity in the context of post-surgical inflammation may result in the targeting of breast cancer cells with stem-like phenotypes, eventually leading to successful suppression of cancer local relapse.

## DISCUSSION

Although the molecular mechanisms linking inflammation and cancer have long remained elusive, a role for inflammation in tumorigenesis is generally accepted. Also the process of wound healing to replace damaged tissue has been linked to cancer. Wound healing is a complex response that includes inflammation, neovascularization, matrix deposition and re-organization and involves a small population of replenishing stem cells giving rise to differentiated progeny that replace the damaged tissue. This characteristic is shared with the stem-like component of a growing tumor and in fact, epithelial tumors have been described as ‘wounds that do not heal’ because of the molecular and cellular similarities between features associated with wounds and carcinomas. Surgery represents an event that acutely causes both inflammation and wound healing response, strongly suggesting that it may represent a perturbing factor in the process of local recurrence or metastasis development in humans [4, 24, 25]. A recent clinical study, reporting that women with T1-2N0 triple-negative breast cancers treated with breast-conserving surgery followed by radiation therapy had significantly better loco-regional control compared with matched women treated with total mastectomy, strongly support these observations [26]. General message that can be drawn is that after extensive mastectomy, an excessive wound response during the healing process may stimulate the secretion of an as-yet uncharacterized growth factor that precipitates loco-regional recurrence. These remarks indirectly confirm our [7, 16, 17] and others' [27, 28] observations, pointing to the wound healing response after surgery as a driving force for the generation of local recurrences.

Many data have established a pivotal role for STAT3 during breast cancer onset and progression [15, 19, 29, 30]. Here, using a large panel of BC cells corresponding to different pathological subtypes, we demonstrate that post-surgical wound fluids induce a highly specific and sustained STAT3 activation (Figure 2). More importantly, we demonstrate that post-surgical wound fluids contain factors that induce the enrichment of breast cancer cells with stem-like and tumor-initiating phenotypes (Figure 1). All tested BC cell lines responded to WF with an increase of mammosphere formation and self-renewal. These phenotypes relied on STAT3 signaling since cells inhibited or silenced for STAT3 lost their stem-like features and high tumorigenicity in tumor-take rate experiments (Figure 4 and 6A and B). It is interesting to note that the same results were not achieved using an antibody able to block IL-6 signaling (Figure 4). IL-6 is often upregulated in epithelial cancers, such as breast and prostate [31, 32] and the IL-6/STAT3 axis is often considered as part of a positive regulatory circuit operating in inflammatory setting to nourish cancer cells and allow to maintain their transformed phenotypes [15]. However, blocking IL-6 signaling alone in our model elicited only a modest decrease in the mammosphere formation, not comparable to the suppression obtained with STAT3 inhibition. Although we know that IL-6 is present and active in the WF [7], these results indicate that it does not represent the principal mediator of STAT3 activation in this setting nor is it the only and/or principal cytokine mediating the WF ability to stimulate stem-like phenotypes in BC cells. Our study suggest that such STAT3-dependent cellular changes may allow the BC cells to compete with the surrounding microenvironment to overcome anti-tumorigenic pressures. Then, the altered microenvironment and the accompanying inflammation become themselves potent tumor promoters, eventually allowing and/or inducing local re-growth. Accordingly, our experiments using a mouse model of BC [16] indicate that silencing STAT3 activity partially prevents formation of local recurrence (Figure 6D and E).

Aberrant activation of STAT3 is commonly found in a large variety of tumors, including breast cancer. Using *in vivo* claudin-low cell line xenograft models of human breast cancer, a very recent study has directly and functionally linked STAT3 signaling activity to breast TIC cell functions [9]. Consequently, STAT3 may be considered a promising target in the field of cancer therapy and, also, for stem-cell-directed therapy in some breast cancer subtypes [9]. We suggest here that for its inhibition to result in a successful therapeutic approach, the correct definition of a target tumor population and the identification of the best treatment window will be critical. Furthermore, our findings support that focusing on the cross talk between breast TIC and their microenvironment may represent a promising way to better target breast cancer cells with stem-like phenotypes.

## Materials and methods

### Study approval

All animal experiments were reviewed and approved by the CRO Institutional Animal Care and Use Committee and were conducted according to that committee's guidelines.

Wound Fluids (WF) were collected at Centro di Riferimento Oncologico, CRO of Aviano, Italy.

Scientific use of biological material was approved by Ethics Committee of the Centro di Riferimento Oncologico, CRO of Aviano, Italy. Specific informed consent was obtained from all patients.

### Cell culture and generation of stable cell clones

MDA-MB-231 (basal, ER-, PR-, HER2-), MDA-MB-453 (luminal, ER-, PR-, HER2-low), MDA-MB-468 (basal, ER-, PR-, HER2-), MCF-7 (luminal, ER+, PR+, HER2-), BT-474 (luminal, ER-, PR-, HER2+) and SK-BR-3 (luminal, ER-, PR-, HER2+) mammary carcinoma cell lines were obtained from ATCC (LGC Standards) and grown in Dulbecco modified Eagle medium (DMEM, Lonza) supplemented with 10% fetal bovine serum (FBS, SIGMA).

STAT3-silenced mammary carcinoma cells were generated by lentiviral transduction of pLKO vectors encoding for human shRNAs of the MISSiON system (sh2_TRCN0000020842, sh3_TRCN0000020843, SIGMA).

All cell lines were authenticated by BMR Genomics srl Padova, Italia, on January 2012 according to Cell ID ^™^ System (Promega) protocol and using Genemapper ID Ver 3.2.1, to identify DNA STR profiles.

### Preparation of protein lysates and immunoblotting analysis

MDA-MB-231, MDA-MB-453, MDA-MB-468, MCF-7, BT-474 and SK-BR-3 mammary carcinoma cell lines were serum starved in DMEM containing 0.1% bovine serum albumin (BSA, SIGMA) and then stimulated with 10% FBS or 5% WF for the indicated time points.

The preparation of protein lysates and immunoblotting analysis was performed as previously described [33], except that membranes were blocked with Odyssey Blocking Buffer (Licor, Biosciences) and, following incubation with primary antibodies overnight at 4°C, incubated 1 hour at RT with IR-conjugated (Alexa Fluor 680, Invitrogen or IRDye 800, Rockland) secondary antibodies for infrared detection (Odyssey Infrared Detection System, Licor).

Primary antibodies directed against AKT (sc-1618), ERK1 (sc-94), STAT3 (sc-482), Fibrillarin (sc-25397) and Vinculin (sc-7694) were purchased from Santa Cruz Biotechnology, Inc.; pT202/204 ERK1/2 (#9101), pS473 AKT (#4060), pY705STAT3 (#9131) were purchased from Cell Signaling; Tubulin (#T9026) was purchased from SIGMA; Cyclin D1 (#04-1151) and Bcl2 (#OP60) were purchased from Millipore; Grb2 was purchased from BD Transduction Laboratories (#610112).

### Separation of nuclear and cytoplasmic fraction

MDA-MB-231 and MDA-MB-468 mammary carcinoma cell lines were serum starved and then stimulated with 5% WF for the indicated time points. Where indicated, cells were pre-treated with the following inhibitors: S3I-201 (Santa Cruz Biotechnology, Inc.) at 50μM or 100μM with pre-treatment of 24hrs, in MDA-MB-231 and MDA-MB-468, respectively; STA-21 (Santa Cruz Biotechnology, Inc.) at 30μM with pre-treatment of 48hrs, in MDA-MB-231 and MDA-MB-468; Stattic (Santa Cruz Biotechnology, Inc.) at 10μM with pre-treatment of 3hrs in MDA-MB-231, at 10μM with pre-treatment of 16hrs in MDA-MB-468; Galiellalactone (Santa Cruz Biotechnology, Inc.) at 12 μM with pre-treatment of 3hrs in MDA-MB-231, 25 μM with pre-treatment of 24hrs in MDA-MB-468. The differential extraction of cytoplasmic and nuclear proteins was performed as previously described [34].

### Wound fluid collection

Drainage Wound Fluids (WF) were collected over the 24hrs after surgery from unselected patients undergone breast-conserving surgery, as described previously [7]. The assays were then performed using a pool of all fluids.

### Proliferation assays

For growth curve, 5×10^4^ cells/well were seeded in 6-well plates in complete medium or in serum free medium supplemented with 3% WF (SFM-3% WF), in triplicate. Where indicated, S3I-201 (Santa Cruz Biotechnology, Inc.; MDA-MB-231: 50μM; MDA-MB-468: 100μM) or STA-21 (Santa Cruz Biotechnology, Inc., MDA-MB-231 and MDA-MB-468: 30μM) were added to the medium. Fresh medium, with or without inhibitors, was changed every other day. At the indicated times, cells were detached with trypsin-EDTA and counted by Trypan Blue exclusion test.

### RNA extraction and qRT-PCR

RNA was extracted using TRIzol (Invitrogen) and was quantified and retro-transcribed with AMV Reverse Transcriptase (Promega) to obtain cDNAs. Absolute expression of human Bcl-2, Survivin and Cyclin D1 was evaluated by qRT-PCR, as previously described [35]. Primers (SIGMA) sequences were as follows:

Bcl-2 FW, 5-TCCGATCAGGAAGGCTAGAGTT-3';

Bcl-2 RW, 5'-CGGTCTCCTAAAAGCAGGC-3'

Survivin FW: 5'-CCACCGCATCTCTACATTCA-3'

Survivin RW: 5'-TATGTTCCTCTATGGGGTCG-3'

Cyclin D1 FW: 5'-AGAAGGAGGTCCTGCCGTCC-3'

Cyclin D1 RW: 5'-GGTCCAGGTAGTTCATGGCC-3'

SDHA FW: 5'-AGAAGCCCTTTGAGGAGCA-3'

SDHA RW: 5'-CGATTACGGGTCTATATTCCAG-3'

GAPDH FW: 5'-GAAGGTGAAGGTCGGAGTC-3'

GAPDH RW: 5'-GAAGATGGTGATGGGATTTC-3'

### Side population analysis

MCF-7 or MDA-MB-468 cells were plated for 48 hours in complete medium or in serum free medium supplemented with 5% WF, as indicated. To identify side population, cells were harvested and resuspended in DMEM 2%FBS, 10mM HEPES, in the presence or not of ABC transporter inhibitor, Reserpine (SIGMA, 50μM) and were incubated for 5 minutes at 37°C. Then, Hoechst-33342 (SIGMA, 5μg/ml) was added to cells for 90 minutes at 37°C. After staining, cells were washed in HBSS 1X, 2%FBS, 10mM HEPES. 7-AAD (2 μg/ml) was added for 10 minutes before FACS analysis, to allow the discrimination of dead versus live cells. Sorting of cells was performed using a FACS LSR Fortessa (Becton Dickinson). Hoechst-33342 dye was excited at 357 nm and its fluorescence was dual-wavelength analyzed (blue, 402–446 nm; red, 650–670 nm).

### Flow cytometric analysis of CD44 and CD24 surface markers in breast cancer cells

MDA-MB-231 were plated for 48 hours in complete medium or in serum free medium supplemented with recombinant epidermal growth factor (rhEGF, 20 ng/ml; SIGMA) or with 5% WF, in the presence or not of STAT3 inhibition. Cells were harvested, washed in PBS 1X and single-stained or double-stained with PE-CD24 (BD Biosciences #555428) and FITC-CD44 (BD Biosciences #347943) antibodies, for 20 minutes at RT. Cells were washed in PBS 1X and analyzed by flow cytometry (FACS LSR Fortessa, Becton Dickinson) to detect levels of surface markers CD44 and CD24.

### Mammospheres Assay

To establish primary mammospheres, cells were plated on poly-HEMA coated dishes as single cell suspension in standard mammosphere medium containing phenol red-free DMEM/F12 (GIBCO), B27 supplement (50x, no vitamin A; Life Techonologies) and recombinant epidermal growth factor (rhEGF, 20 ng/ml; SIGMA). Where indicated, cells were plated in medium containing phenol red-free DMEM/F12, B27 supplement and 5% WF. In a subset of experiments, blocking antibody anti-IL6 (R&D Systems, 0.2μg/ml) or STAT3 inhibitors (S3I-201; STA-21; Stattic; Galiellalactone, purchased from Santa Cruz Biotechnology, Inc.) were added to the medium. After ten days, primary mammospheres were counted. To establish secondary mammospheres, primary mammospheres were collected, disaggregate in trypsin using 25-gauge needle fitted to a syringe. Cells were plated at the same seeding density of the primary generation. Mammosphere forming efficiency (MFE%) was calculated as follows: number of mammospheres per well/number of cells seeded *per* well × 100. Mammosphere self-renewal was calculated as follows: total number of secondary mammospheres /total number of primary mammospheres.

### Immunoassay for the detection of phosphorylation of STAT proteins

The detection of the activation of STAT phospho-protein was performed using the Milliplex Map 5-Plex Human STAT Phosphoprotein Magnetic Bead Kit (Millipore). MDA-MB-231 cells were starved in DMEM containing 0.1% BSA and stimulated or not with WF 5% for 20 minutes and processed as indicated by the manufacturer's instruction.

### *In vivo* experiments

Primary tumors were established by injection of 1×10^6^ MDA-MB-231 control or 2×10^6^ MDA-MB-231 shSTAT3-derived cell clones, bilaterally in the fat pads of the thoracic mammary glands of female athymic nude mice (Harlan, 6-8 weeks old). Growth of primary tumors was monitored by measuring tumor length (L) and width (W) and calculating tumor volume, based on the formula L x W^2^ / 2. The evaluation of local relapse was performed as previously described [16]. To analyze the tumor take rate, mice were injected with 1×10^4^ or 2×10^4^ or 5×10^4^ or 1×10^5^ or 2×10^5^ or 2×10^6^ MDA-MB-231 control or shSTAT3 cells, resuspended in 50 μl Matrigel/PBS (1:1). Growth of primary tumors was monitored for up to 12 weeks.

### Statistical analyses

Data were examined using the two-tailed Student t test or unpaired two-tailed Mann-Whitney U test. Differences were considered significant at p < 0.05. The computer software PRISM (version 4, GraphPad, Inc.) was used to make graphs and all statistical analyses.

### Competing interests

The authors declare that they have no competing interests.

Supplementary Information accompanies this paper on the Oncotarget website.

## SUPPLEMENTARY MATERIAL FIGURES


