# Supplementary material for: Surgery-induced wound response promotes stem-like and tumor-initiating features of breast cancer cells, via STAT3 signaling
Source: Oncotarget. 2014 Jul 9;5(15):6267–79. doi: 10.18632/oncotarget.2195 (PMC4171628; doi:10.18632/oncotarget.2195)
Supplement: Supplementary file 1 [file oncotarget-05-6267-s001.pdf]

Surgery-induced wound response promotes stem-like and tumor-initiating features of breast cancer cells, *via* STAT3 signaling

Supplementary Material

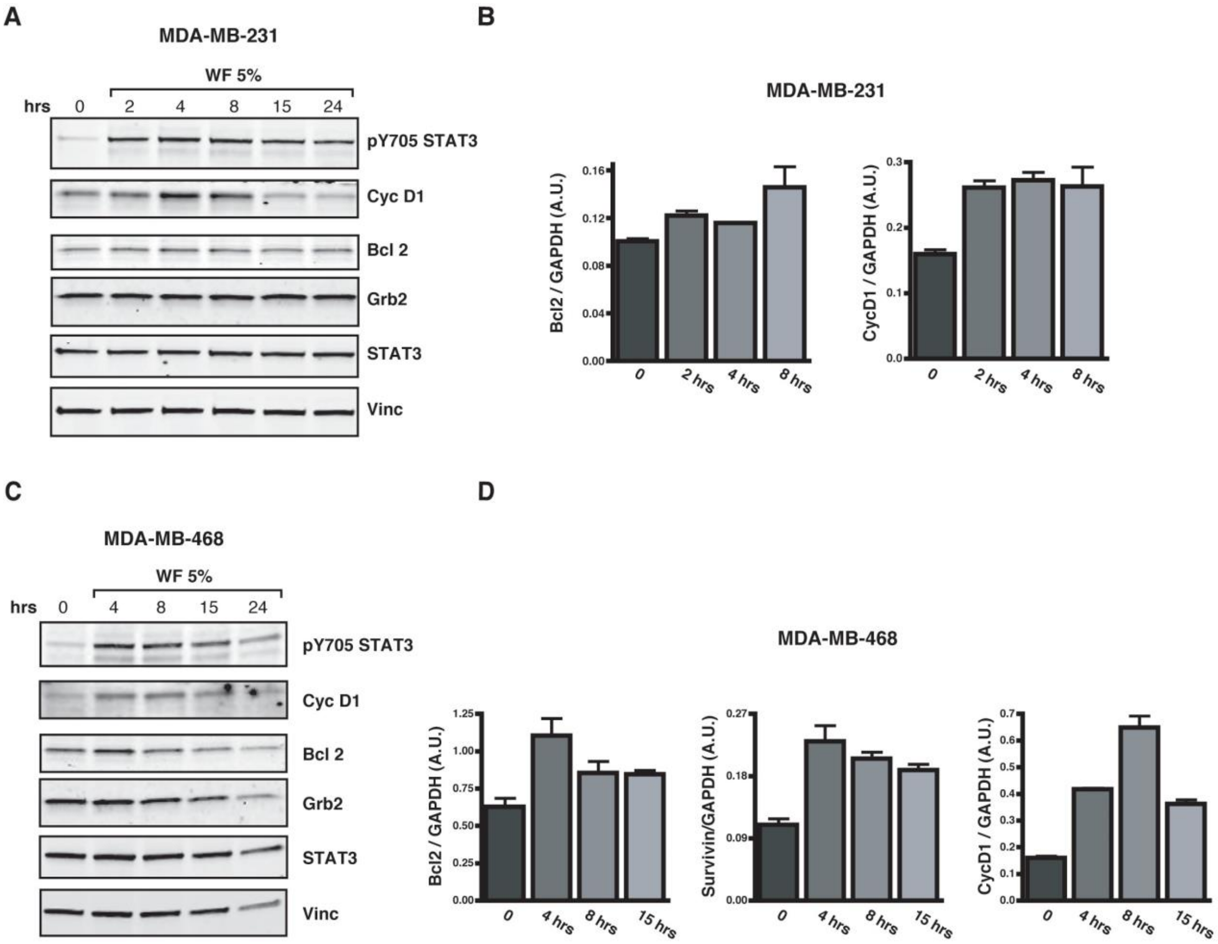

Supplemental Figure S1. Breast cancer cell stimulation with WF elicits sustained activation of STAT3 signaling pathway. (**A**) Western blot analysis of MDA-MB-231 cells serum starved and then stimulated for the indicated times with 5% wound fluids (WF). Grb2 and vinculin expression were used as loading control. (**B**) qRT-PCR analysis of Bcl-2 and Cyclin D1 expression in MDA-MB-231 cells serum starved and then stimulated for the indicated times with 5% WF. Data represents the mean (± S.D.) of two independent experiments performed in triplicate. (**C**) Same as in (A) but using MDA-MB-468 cells. (**D**) Same as in (B) but using MDA-MB-468 cells and analyzing the expression of Bcl-2, Survivin and Cyclin D1.

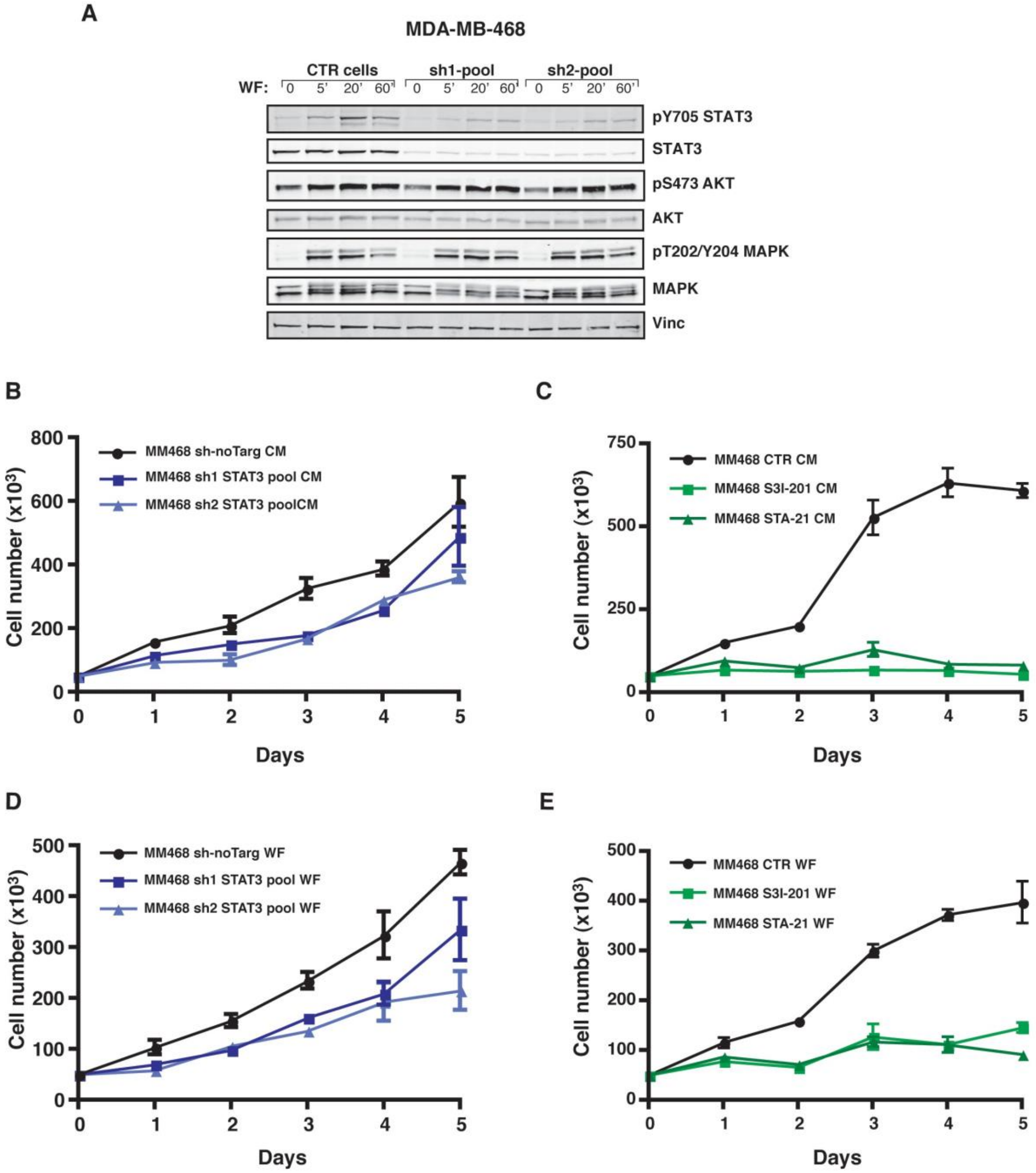

Supplemental Figure S2. Activation of STAT3 following WF stimulation is efficiently impaired in breast cancer cells modified for STAT3 expression. (**A**) Western blot analysis of MDA-MB-468 cell line stably transduced with a lentiviral vector encoding for control sh-RNA (CTR) or for sh-RNAs directed against human STAT3 (sh), serum starved and then stimulated

for the indicated times with 5% wound fluids (WF). Vinculin expression was used as loading control. (**B**) Growth curve analysis of MDA-MB-468 control cells (sh-no Target) or STAT3 silenced clones (sh-STAT3). Cells ($50x10^3$/well) have been seeded on day 0, in complete medium (CM) as indicated, and then counted by Trypan blue exclusion test, every day for 5 days. Two independent cell clones have been evaluated. Data represents the mean (± S.D.) of two independent experiments performed in triplicate. (**C**) Same as in (B), but seeding cells in serum free medium supplemented with 3% wound fluids (WF). Two independent cell clones have been evaluated. Data represents the mean (± S.D.) of two independent experiments performed in triplicate. (**D**) Growth curve analysis of MDA-MB-468 cell line in the presence of the indicated inhibitors. Cells ($50x10^3$/well) have been seeded in complete medium (CM) on day 0, in the presence of S3I-201 (100 µM) or STA-21 (30 µM) or vehicle (CTR) and then counted by Trypan Blue exclusion test, every day for 5 days. Fresh medium containing the inhibitor was replaced on day 3. Data represent the mean (± S.D.) of two independent experiments performed in triplicate. (**E**) Same as in (D), but seeding cells in serum free medium supplemented with 3% wound fluids (WF). Data represents the mean (± S.D.) of two independent experiments performed in triplicate.

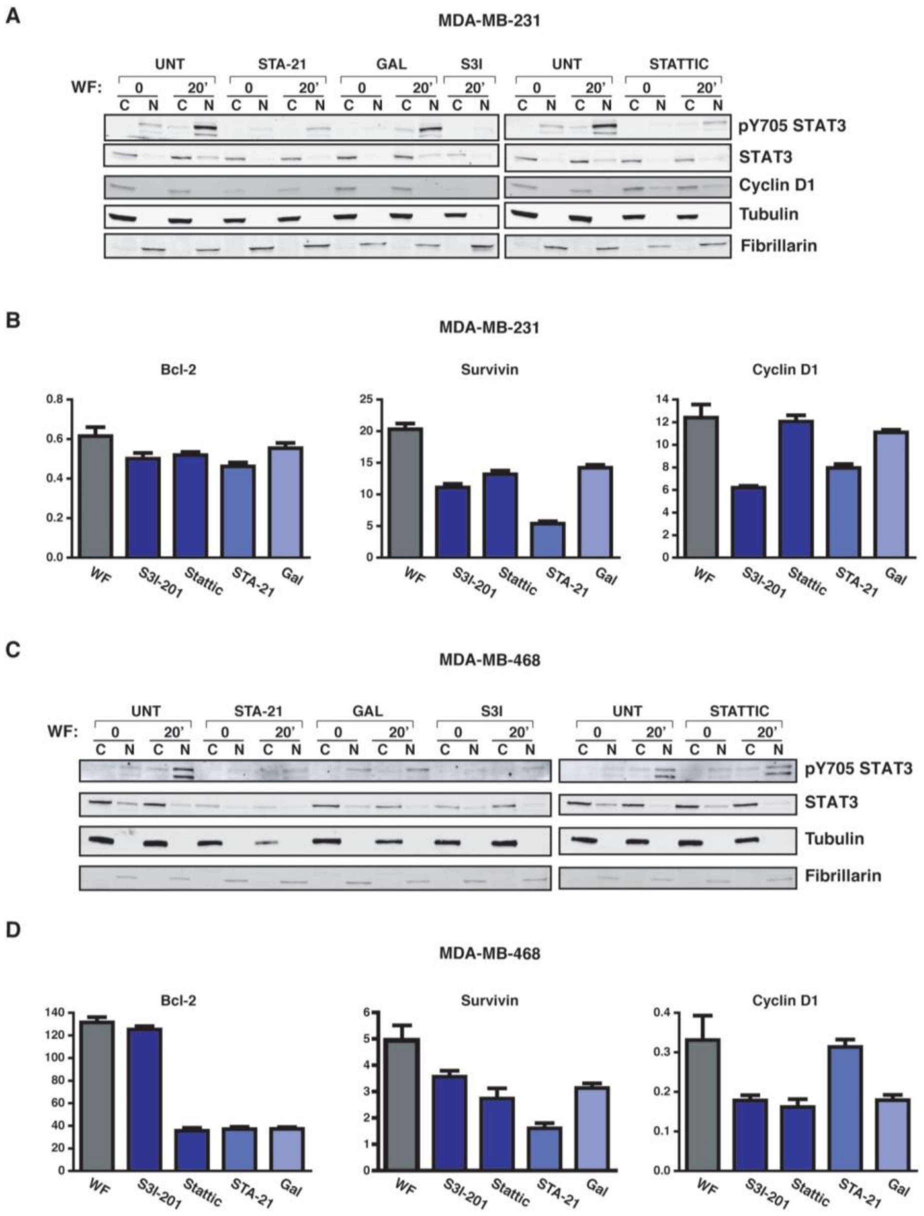

Supplemental Figure S3. Activation of STAT3 following WF stimulation is efficiently impaired in STAT3 inhibited breast cancer cell lines. (**A**) Western blot analysis of cytoplasmic and nuclear fraction of MDA-MB-231 cells, serum starved and harvested at time 0 or stimulated for 20 minutes with 5% wound fluids (WF), with or without STAT3 inhibitors (STA-21, 30 µM; Galiellalactone, 12 µM; S3I-201, 50 µM; and Stattic, 10 µM). Tubulin was used as cytoplasmic marker, fibrillarin as nuclear marker. (**B**) qRT-PCR analysis of Bcl-2,

Survivin and Cyclin D1 expression in MDA-MB-231, stimulated with WF and treated with STAT3 inhibitors (S3I-201, 50 µM; Stattic, 10 µM; STA-21, 30 µM; and Galiellalactone, 12 µM) or left untreated (WF). (**C**) Western blot analysis of cytoplasmic and nuclear fraction in MDA-MB-468 cells, serum starved and harvested at time 0 or stimulated for 20 minutes with 5% wound fluids (WF), with or without STAT3 inhibitors (STA-21, 30 µM; Galiellalactone, 25 µM; S3I-201, 100 µM; and Stattic, 10 µM). Tubulin was used as cytoplasmic marker, fibrillarin as nuclear marker. (**D**) Same as in (B), but in MDA-MB-468 cells.

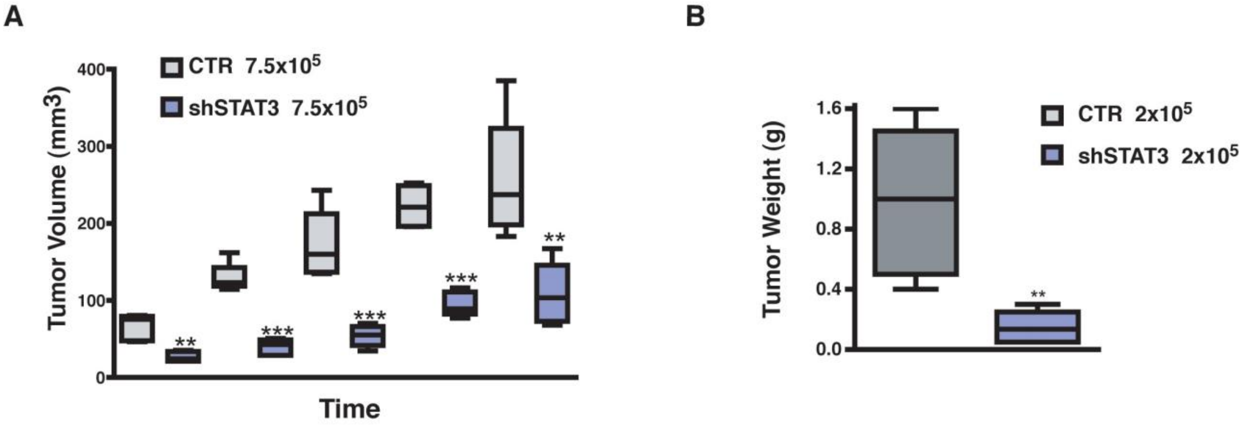

Supplemental Figure S4. Growth of primary breast tumors is significantly suppressed by silencing STAT3 expression. (**A**) Graph reports the volume (mm^3) of primary tumors derived from injection of $7.5x10^5$ MDA-MB 231 control (CTR) or STAT3 silenced (sh-STAT3) cells in thoracic mammary fat pads of nude mice (2 MFP/mouse) in 50 µl Matrigel/PBS (1:1). (**B**) Graph reports the tumor weight of the experiment described in Figure 5D. One asterisk (*) indicates a p value ≤ 0.05, two asterisks (**) a p value ≤ 0.01 and three asterisk (***) a p value ≤ 0.005.
